# Altered physiological brain variation in drug‐resistant epilepsy

**DOI:** 10.1002/brb3.1090

**Published:** 2018-08-15

**Authors:** Janne Kananen, Timo Tuovinen, Hanna Ansakorpi, Seppo Rytky, Heta Helakari, Niko Huotari, Lauri Raitamaa, Ville Raatikainen, Aleksi Rasila, Viola Borchardt, Vesa Korhonen, Pierre LeVan, Maiken Nedergaard, Vesa Kiviniemi

**Affiliations:** ^1^ Department of Diagnostic Radiology Medical Research Center Oulu University Hospital Oulu Finland; ^2^ Oulu Functional NeuroImaging‐Group Research Unit of Medical Imaging, Physics and Technology University of Oulu Oulu Finland; ^3^ Research Unit of Neuroscience, Neurology University of Oulu Oulu Finland; ^4^ Department of Neurology and Medical Research Center Oulu Oulu University Hospital Oulu Finland; ^5^ Department of Clinical Neurophysiology Medical Research Center Oulu Oulu University Hospital Oulu Finland; ^6^ Faculty of Medicine Department of Radiology – Medical Physics University Medical Center Freiburg University of Freiburg Freiburg Germany; ^7^ Center for Translational Neuromedicine Department of Neurosurgery University of Rochester Rochester New York; ^8^ Faculty of Health and Medical Sciences Center for Basic and Translational Neuroscience University of Copenhagen Copenhagen Denmark

**Keywords:** brain physiology, coefficient of variation, epilepsy, fMRI, MREG, multimodal imaging

## Abstract

**Introduction:**

Functional magnetic resonance imaging (fMRI) combined with simultaneous electroencephalography (EEG‐fMRI) has become a major tool in mapping epilepsy sources. In the absence of detectable epileptiform activity, the resting state fMRI may still detect changes in the blood oxygen level‐dependent signal, suggesting intrinsic alterations in the underlying brain physiology.

**Methods:**

In this study, we used coefficient of variation (CV) of critically sampled 10 Hz ultra‐fast fMRI (magnetoencephalography, MREG) signal to compare physiological variance between healthy controls (*n* = 10) and patients (*n* = 10) with drug‐resistant epilepsy (DRE).

**Results:**

We showed highly significant voxel‐level (*p* < 0.01, TFCE‐corrected) increase in the physiological variance in DRE patients. At individual level, the elevations range over three standard deviations (*σ*) above the control mean (*μ*) CV_MREG_ values solely in DRE patients, enabling patient‐specific mapping of elevated physiological variance. The most apparent differences in group‐level analysis are found on white matter, brainstem, and cerebellum. Respiratory (0.12–0.4 Hz) and very‐low‐frequency (VLF = 0.009–0.1 Hz) signal variances were most affected.

**Conclusions:**

The CV_MREG_ increase was not explained by head motion or physiological cardiorespiratory activity, that is, it seems to be linked to intrinsic physiological pulsations. We suggest that intrinsic brain pulsations play a role in DRE and that critically sampled fMRI may provide a powerful tool for their identification.

## 1 INTRODUCTION

Epilepsies are a diverse group of brain diseases manifesting themselves as repetitive seizure activity (Fisher et al., 2014), with a life‐time prevalence of 7.6/1,000 persons (Fiest et al., 2017). Cohort studies have shown that 30%–40% of epilepsies are drug resistant (Brodie, Barry, Bamagous, Norrie, & Kwan, 2012; Elger & Schmidt, 2008; Laxer et al., 2014; Pitkänen et al., 2016). In anatomical brain scans of patients with drug‐resistant epilepsy (DRE), mesial temporal sclerosis (MTS) has been detected in 40%–50% of the patients (Jutila et al., 2002; Lapalme‐remis & Cascino, 2016), while otherwise the causes of epilepsy fell to other etiologic categories.

For epilepsy patients, functional magnetic resonance imaging (fMRI) has been used to localize language areas in the brain via task activations studies and imaging at resting state as well as to evaluate surgical outcomes (Chaudhary, Duncan, & Lemieux, 2013; Constable et al., 2013; van Graan, Lemieux, & Chaudhary, 2015; Lee, Smyser, & Shimony, 2013; Pittau, Ferri, Fahoum, Dubeau, & Gotman, 2017; Proulx et al., 2014; Robinson et al., 2017; Tracy & Doucet, 2015). FMRI combined with simultaneous electroencephalography (EEG‐fMRI) has become a useful tool in mapping frequent epileptiform activity in the form of spikes and seizure activity (Abela et al., 2013; Gotman, 2008; Jacobs et al., 2009; Moeller et al., 2009; Pittau et al., 2017).

A recent advance in the field is the utilization of ultra‐fast fMRI sequence such as magnetic resonance encephalography (MREG), which enables a physiologically accurate 10 Hz image sampling rate without aliasing of the cardiorespiratory activity over low‐frequency activity (Assländer et al., 2013). This methodology enables robust mapping of epileptic spike activity as detected in simultaneous EEG‐fMRI due to the increased temporal accuracy and statistical power (Jacobs et al., 2014). MREG can also discern three different forms of physiological brain pulsations; cardiac pulses, respiratory oscillations, and very low‐frequency fMRI signal fluctuations (Kiviniemi et al., 2016). These are important for the detection of driving forces of recently detected glymphatic brain clearance mechanisms, which have been linked to several neurological diseases (Sun et al., 2017).

In the absence of frequent (inter‐) ictal activity, EEG, fMRI, MEG, or their simultaneously acquired combinations, fail to detect abnormality in a majority of epilepsy patients, whereas resting state fMRI has been able to detect altered functional connectivity and regional homogeneity of the blood oxygen level‐dependent (BOLD) signal in epilepsy patients (Constable et al., 2013; Lee, Smyser, et al., 2013; Mankinen et al., 2011, 2012; Wurina, Zang, & Zhao, 2012). In focal epilepsy, functional connectivity could be altered even outside the epileptogenic region (Tracy & Doucet, 2015). Most of these studies have been performed under the assumption that the control and patient populations share the common Gaussian BOLD signal noise distribution.

This assumption of identical signal distributions may not be true, since recently BOLD signal noise characteristics have been shown to be altered by disease (Khalil et al., 2017; Makedonov, Black, & MacIntosh, 2013; Makedonov, Chen, Masellis, & MacIntosh, 2016; Tuovinen et al., 2017). A recently emerged metric to assess BOLD signal properties is the coefficient of variation (CV), which has previously been used to reflect stability of a measured process. CV is sensitive to subtle changes in signal characteristics of the BOLD data (Jahanian et al., 2014) and can provide a quality assurance metric (Tuovinen et al., 2017). Interestingly, CV has also been shown to reflect changes in physiological fluctuations in BOLD signal in white matter. Moreover, altered values of CV have been detected in acute ischemic stroke (Khalil et al., 2017), Alzheimer's disease (Makedonov et al., 2016; Tuovinen et al., 2017), and small vessel disease (Makedonov et al., 2013).

Aliasing of physiological pulsations over slow ones reduce the accuracy of CV noise variance and prevents the identification of the noise source. Fast fMRI sequences like MREG enable separation of physiological noise sources in the absence of aliasing due to critical sampling. Furthermore, the CV noise variance measures are more accurate from the larger signal distributions having thousands of samples. Therefore, CV is a promising metric to analyze BOLD signal properties derived using MREG.

Using the latest developments, we compared physiological noise in DRE patients to matched control groups especially on concentrating on individual changes in this study. The null hypothesis was that the physiological signal variation as measured with the CV of ultra‐fast fMRI signal should be identical in DRE patients and matched controls.

## 2 METHODS

### 2.1 Participants

The study sample consisted of 10 patients with DRE (age 34.5 ± 10.9 years, seven females, Table 1) and 10 age‐ and sex‐matched healthy controls (HC, age 34.4 ± 11.3 years, seven females). We included also another control group for verification purposes (HC_2nd_, 10 subjects, age 23.7 ± 2.2 years, eight males). The patients were recruited from the outpatient department of neurology at Oulu University Hospital. The patients were diagnosed with focal epilepsy with ongoing seizure activity despite the use of two to four antiepileptic drugs. The patients either had been or were to be evaluated for the possibility of epilepsy surgery or electrical stimulation treatments. The mean age at epilepsy onset was 24.2 ± 12.8 years. Subjects were scanned between 2012 and 2016.

**Table 1:** Clinical characteristics of 10 recruited epilepsy patients

DRE patient	Duration of epilepsy, years	Seizure type	Previous neurophysiological findings	Antiepileptic drugs
1	8	Focal with impaired awareness	MRI: Previously normal. Now, right amygdala/temporal cortex unspecified pathology EEG: Normal PET: Left temporal hypometabolism, mild right temporal hypometabolism	Carbamazepine 800 mg, Pregabalin 300 mg
2	10	Focal with impaired awareness	MRI: Mild right hippocampal sclerosis EEG: Normal	Lamotrigine 550 mg, Topiramate 200 mg
3	19	Focal to bilateral tonic–clonic	MRI: Normal EEG: Bilateral epileptiform discharges with possible frontal onset	Lacosamide 200 mg, Lamotrigine 175 mg, Valproic acid 1500 mg, Clonazepam 10 mg
4	4	Focal with impaired awareness	MRI: Normal, possible FCD in left temporal middle gyrus EEG: Left frontotemporal epileptiform discharges	Lacosamide 150 mg Zonisamide 100 mg
5	6	Focal to bilateral tonic–clonic	MRI: Normal EEG: Interictal epileptiform discharges in the left parieto‐occipital area without constant local findings.	Levetiracetam 2,500 mg, Oxcarbazepine 1,200 mg, Lacosamide 300 mg
6	13	Focal to bilateral tonic–clonic	MRI: Normal EEG: Normal	Lamotrigine 100 mg, Lacosamide 200 mg, Topiramate 125 mg
7	12	Focal to bilateral tonic–clonic	MRI: Normal EEG: Single spike and slowing in right temporobasal and posterior temporal area during hyperventilation	Valproic acid 900 mg, Lamotrigine 250 mg, Clonazepam 10 mg
8	11	Focal with impaired awareness	MRI: Normal EEG: Ictal onset left temporal PET: Left temporal hypometabolism	Oxcarbazepine 1,200 mg, Zonisamide 400 mg
9	12	Focal with impaired awareness	MRI: Normal EEG: Ictal onset right posterior fusiform gyrus	Topiramate 100 mg, Lacosamide 500 mg, Clonazepam 30 mg
10	7	Focal with impaired awareness	MRI: Normal EEG: Ictal onset left temporal	Levetiracetam 2,000 mg, Lacosamide 150 mg, Lamotrigine 400 mg

*Note*

DRE: drug‐resistant epilepsy; MRI, magnetic resonance imaging; EEG: electroencephalography.

Written informed consent was obtained from all of the participants according to the Declaration of Helsinki. The Ethics Committee of the Northern Ostrobothnia Hospital District, Finland, approved the research protocol.

### 2.2 Multimodal data acquisition

Multimodal imaging was carried out using our Hepta‐scan concept, in which fast fMRI data was imaged simultaneously and synchronously with EEG, noninvasive blood pressure (NIBP), and near‐infrared spectroscopy (NIRS), and anesthesia monitor data (Korhonen et al., 2014). We also utilized the MRI scanner′s own respiratory bellows and peripheral capillary oxygen saturation meters. All data were synchronized with the scanner trigger‐pulse. Anesthesia monitor data were used for verification purposes.

### 2.3 Functional MR imaging

All subjects were scanned using a Siemens MAGNETOM Skyra 3T MRI scanner (Siemens Healthcare GmbH, Germany) with a 32‐channel head coil. We utilized a MREG sequence (Assländer et al., 2013; Lee, Zahneisen, Hugger, LeVan, & Hennig, 2013; Zahneisen et al., 2012), which includes a three‐dimensional (3D) single‐shot undersampled spiral trajectory with the following sequence parameters: repetition time (TR) = 100 ms, echo time (TE) = 36 ms, field of view (FOV) = 192, matrix 64^3^, 3‐mm cubic voxel, and flip angle = 25°. MREG data were reconstructed by L2‐Tikhonov regularization with lambda = 0.1, with the latter regularization parameter determined by the L‐curve method (Hugger et al., 2011). An analysis of the point‐spread function revealed that the resulting effective spatial resolution was 4.5 mm. This resting state MREG data acquisition lasted 10 min and patients were imaged 2–4 times consecutively with 2–5 min apart, because we intended to capture possible spontaneous epileptiform activity. Because all patients had at least two scans, both of them were further analyzed. HC group were imaged only once and some only for 5 min due to schedule reasons, because epileptiform activity was not to be expected.

Subjects were given ear plugs to reduce noise and soft pads were fitted over the ears to minimize motion. During scanning, all participants received the instructions to simply rest, stay awake, and focus gaze on a cross on a screen, which they saw via a mirror mounted on the head coil. High‐resolution T1‐weighted MPRAGE (TR = 1,900 ms, TE = 2.49 ms, TI = 900 ms, FA = 9°, FOV = 240, slice thickness 0.9 mm) images were obtained for co‐registration of the MREG data to Montreal Neurologic Institute (MNI 152) in 4 mm ISO standard space.

### 2.4 Cardiorespiratory data and electroencephalography data

Anesthesia monitoring signals (PPG, ETCO_2_, ECG, and cuff‐based blood pressure) were also registered using 3T MRI‐compatible anesthesia monitor (GE Datex‐Ohmeda^™^; Aestiva/5 MRI). In some cases, the ETCO_2_ data measured from by the anesthesia monitor were corrupted. Thus, we used scanners inbuilt PPG and respiratory bellows data for groups comparisons.

EEG was recorded with an MR‐compatible BrainAmp system (Brain Products, Gilching, Germany) with 32 Ag/AgCl electrodes (including one ECG electrode) placed according to the international 10–20 system. To get low electrode impedances (<5 kΩ), the skin potential was removed with the stick abrasion technique (Vanhatalo et al., 2003). Data sampling rate was 5 kHz and band pass from DC to 250 Hz. Signal quality was tested outside the scanner room by recording 30‐s eyes open and eyes closed. MR‐scanner optical timing pulse and BrainAmp SyncBox were used to ensure that the EEG and fMRI data were in synchrony. The amplifier was placed outside the bore, and cables were stabilized to avoid motion artifacts.

### 2.5 Data preprocessing

#### 2.5.1 Imaging data

MREG data were preprocessed with the FSL pipeline introduced by (Jenkinson, Beckmann, Behrens, Woolrich, & Smith, 2012). To minimize T1‐relaxation effects, 180 timepoints were removed from the beginning. Data were high‐pass filtered with cutoff frequency of 0.008 Hz (125 s). Head motion was corrected with FSL 5.08 MCFLIRT software (Jenkinson, Bannister, Brady, & Smith, 2002). Brain extraction was carried out with optimization of the deforming smooth surface model, as implemented in FSL 5.08 BET software (Smith, 2002). Spatial smoothing was carried out with *fslmaths* 5‐mm FWHM Gaussian kernel. MREG images were aligned to three‐dimensional (MPRAGE) anatomical images in MNI152 standard space (full‐search, 12 DOF) in 4‐mm resolution. Advanced ICA FIX method was used for secondary artifact removal from the preprocessed MREG data (Griffanti et al., 2014; Salimi‐Khorshidi et al., 2014). FIX method was trained on previously acquired control data and was used for both groups because of our null hypothesis. The first 5‐min data, that is, 3,000 brain volumes were used for the actual CV_MREG_ calculations as in our previous study and to minimize vigilance drops (Kiviniemi et al., 2016). Data from patient 4 were excluded for the first measurement group analysis due to partial data corruption.

The CV_MREG_ maps were calculated from full band (>0.008 Hz) fMRI data and from three sub‐bands. These sub bands were the very low frequency (VLF) 0.009–0.1 Hz, the respiratory frequency 0.12–0.4 Hz, and the cardiac frequency 0.9–1.5 Hz. We chose these bands based on the collected physiological data of controls and patients. With these bandwidths, we could be certain that every subject's physiological pulsations were included in the corresponding band‐pass filtered data. These sub‐bands were filtered from original fMRI data with Analysis of Functional Neuroimages (AFNI) toolbox *3dbandpass* filtering command. After filtering sub‐bands, we added corresponding full band mean image voxel‐wise, because filtering demeans the signal around zero and otherwise CV_MREG_ calculation would be impossible. For display and further analysis, CV_MREG_ maps were interpolated to 2‐mm MNI‐space.

#### 2.5.2 Physiological verification of data

Cardiac PPG (Scanner and Aestiva‐monitor) and respiratory data (Aestiva ETCO_2_ & scanner respiratory bellows) were downsampled to 10 Hz for heart and respiration rate calculation in MatlabR2016b. The downsampling was performed by taking every 30 sample of original signal. With downsampling, we were also able to minimize Siemens 3T SKYRA trigger pulse artifact. If there were still artifact left after downsampling, we removed those single spikes before calculations.

Brain Products 32‐channel EEG pre‐processing was performed offline using Brain Vision Analyzer (version 2.0, Brain Products). Gradient and ballistocardiographic artifact correction was carried out with the average artifact subtraction method (Allen, Josephs, & Turner, 2000; Allen, Polizzi, Krakow, Fish, & Lemieux, 1998) as in our previous studies (Hiltunen et al., 2014; Korhonen et al., 2014).

### 2.6 Coefficient of variation (CV_MREG_) maps

**Figure 1:**
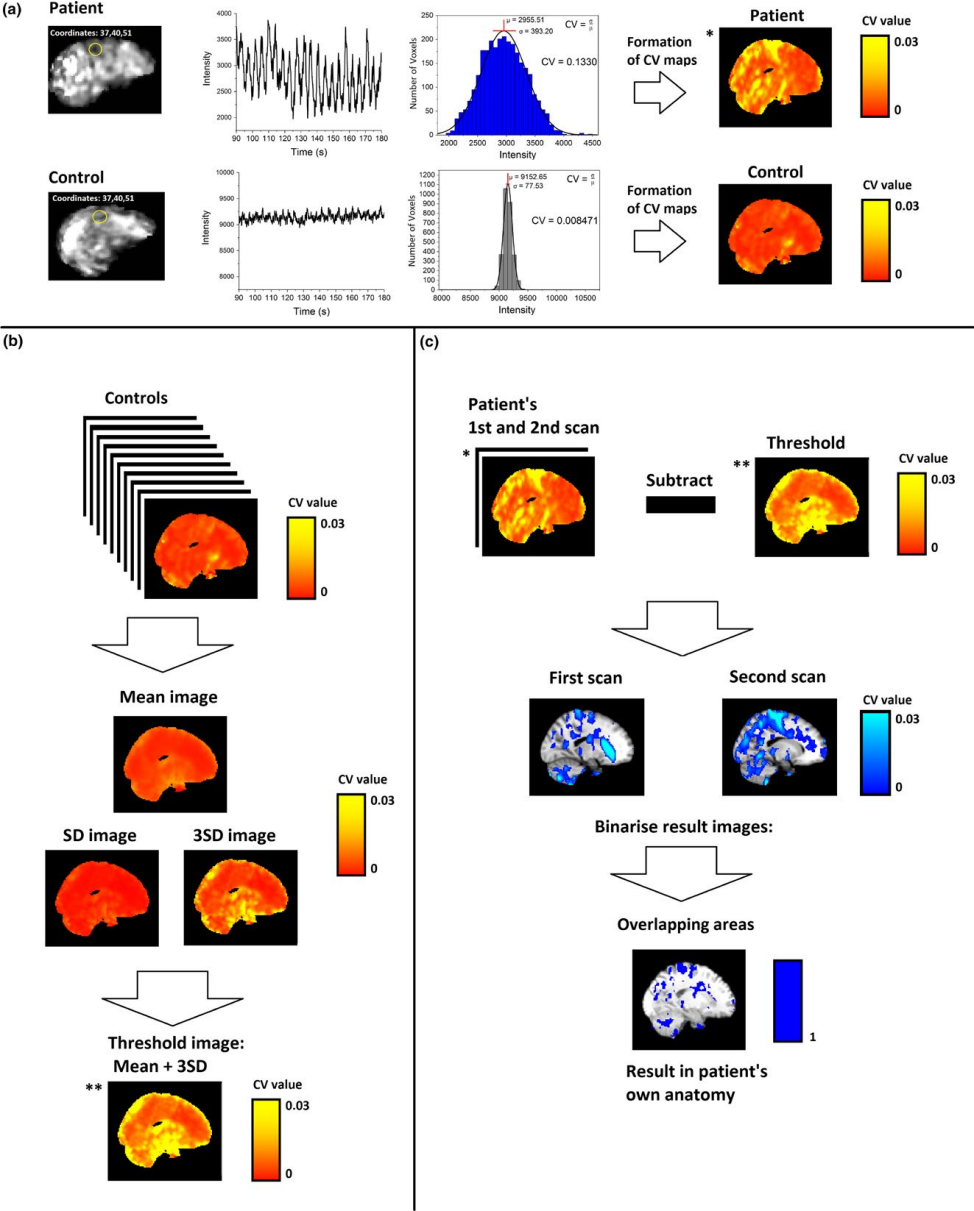
Calculation and analysis of CV_MREG_. (a) Example randomly selected patient and control subject for CV_MREG_ voxel‐wise calculation with an example of signal and it is mean and SD_MREG_ values of single voxel. After voxel‐wise calculation of CV_MREG_ data were normalized to standard MNI152 space and the whole‐brain CV_MREG_ map was produced. (b) Threshold image was derived from HC data by calculating mean and SD_MREG_ within the group. The SD_MREG_ value was multiplied by 3 and added to the HC mean to obtain the threshold image. (c) The previously calculated threshold image from HC group was subtracted from each patient measurement for individual map of CV_MREG_. Values over the threshold were binarized, and then, the overlapping areas in both measurements were calculated. *,** indicates the same image in the calculation process

Figure 1 illustrates the main analysis schema of the study. CV was calculated as follows:

(1) $$\text{CV}=\frac{\sigma (X)}{\mu (X)}$$

where X is signal time series, *μ* is mean, and *σ* is standard deviation (*SD*). For the fMRI data, *fslmaths* was used to calculate voxel‐wise time domain signal mean (*μ*) and *SD* (*σ*) values for every subject. Calculation of CV_MREG_ was executed for both full‐band and filtered data in the same way. Whole brain global signal values were calculated using *fslmeants* for full band MREG data. From this individual mean signal, the signals *μ*,* σ*, CV_MREG_ were calculated. For the physiological verification data (PPG, respiratory waveforms), these variables were calculated in MATLAB.

The normal range of voxel‐wise CV_MREG_ values were calculated within the HC group to produce a voxel‐wise threshold map of CV_MREG_. The threshold voxel‐wise CV map CV_MREG_thr_ was calculated as follows:

(2) $${\text{CV}}_{\mathrm{MREG}}\_\mathrm{thr}=\mu +3\sigma$$

where *μ* is mean value of CV_MREG_ in HC group, and *σ* is its *SD* (Figure 1). This threshold encompassed the normal range of CV values. Individual CV_MREG_ maps in the epilepsy group were then thresholded at CV_MREG_thr_ to identify voxels above this range. As patients had two measurements, the thresholded maps from each measurement were binarized and combined, preserving only the overlapping voxels from both measurements that exceeded CV_MREG_thr_. Thresholding was performed only for full band data, because of the previously mentioned voxel‐wise adding of full‐band mean to filtered data. We visually inspected the corresponding areas exceeding the threshold and compared them to clinical symptoms.

### 2.7 Voxel‐wise analysis between‐group differences in CV maps

The contrasts between the localization of CV_MREG_ values of two groups were analyzed with FSL's *randomize* with 10000 random permutations implanting threshold‐free cluster enhancement (TFCE) correction in both directions (HC > DRE, HC < DRE) separately (Smith & Nichols, 2009). The *t*‐statistic maps with corrected *p*‐values (*p* < 0.05) were created to evaluate statistical significance of the CV_MREG_ maps between the group (Nichols & Holmes, 2002; Winkler, Ridgway, Webster, Smith, & Nichols, 2014). This analysis was performed with full band and filtered bands accordingly.

### 2.8 Template based analysis and global signal

For the template‐based analysis the gray matter (GM), white matter (WM), and cerebrospinal fluid (CSF), masks were created for standard MNI 2 mm space and were used to mask CV maps. The mean values of masked CV_MREG_ values were calculated afterward to compare groups.

The global signal was calculated from masked, with brain only MNI space voxels mean (FSL command: *fslmeants*) MNI space voxels mean. This mean was calculated for every subject over time (5 min) to get global signal over time. The mean and *SD* was calculated from previously acquired global signal for the CV calculation.

### 2.9 Evaluation of potential sources of CV differences

#### 2.9.1 Physiological

Data from the scanner's inbuilt respiration bellows and PPG were visually inspected, and signal frequency, mean, and *SD* were calculated. With mean and *SD*, we calculated the physiological signal CV. Wilcoxon rank‐sum test was used to compare these values between groups.

#### 2.9.2 Motion

Although head motion was corrected with MCFLIRT, absolute and relative movement was also visually inspected. From each motion time series, mean, *SD*, and CV were calculated and compared between groups using Wilcoxon rank‐sum test.

## 3 RESULTS

The EEG data from epilepsy group were checked by a clinical neurophysiologist (SR) for spontaneous epileptiform activity. Clearly identifiable epileptiform activity was not detected in any of the DRE patients nor HC group. EEG data were not further processed or analyzed.

### 3.1 Individual mapping of DRE patients

Thresholding of DRE patients’ CV maps with CV_MREG_thr_ enabled the detection the areas of elevated CV_MREG_ >3*SD* in each patient separately. We further investigated whether the overlapping areas include only voxels that were above the CV_MREG_ limit in ***both*** first and second measurements (CV_MREG_ > *μ* +3*σ*). There were no significant differences between groups and individuals with decreased values (CV_MREG_ < *μ*‐2*σ*).

**Figure 2:**
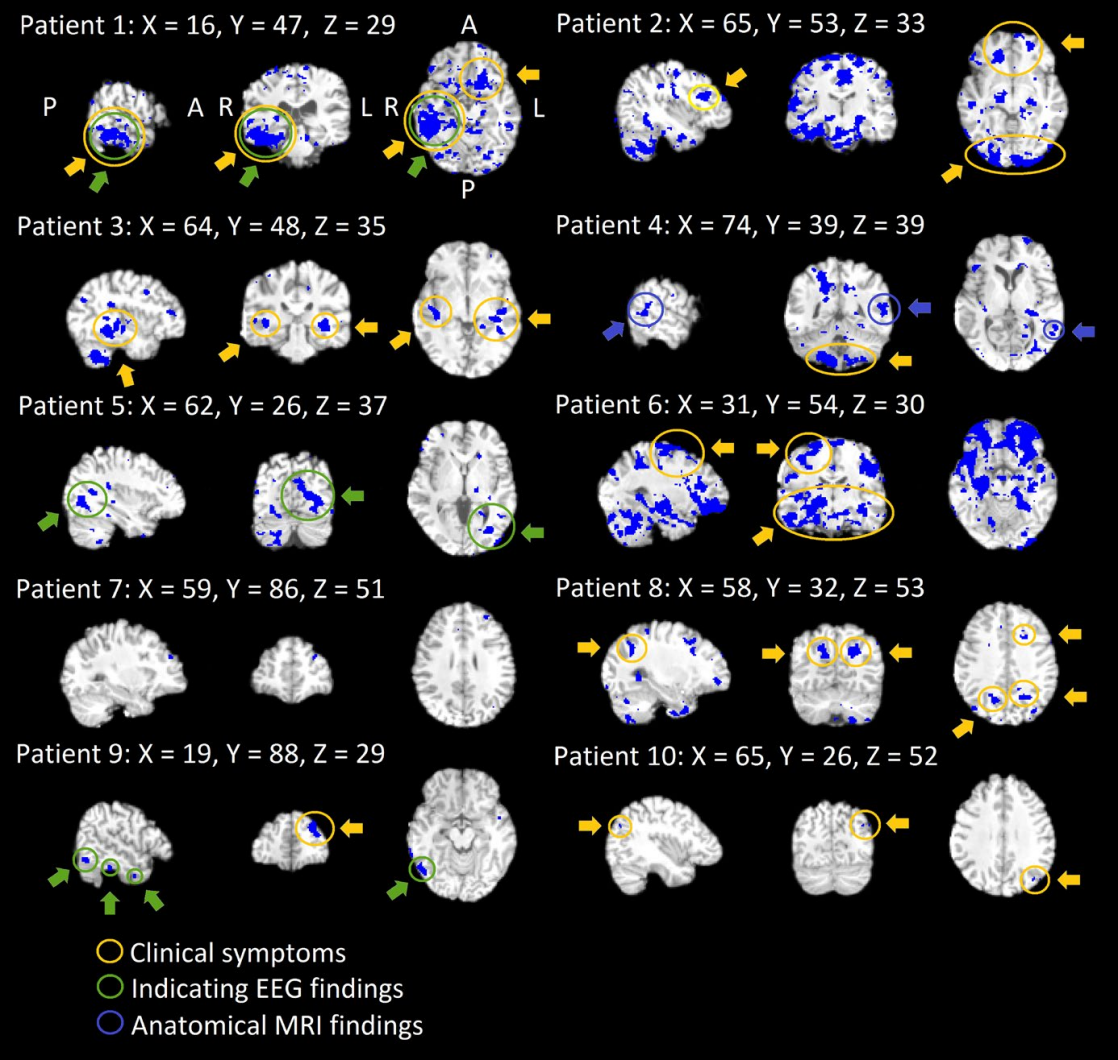
Individual findings after thresholding consecutively. Individual threshold maps of overlapping areas in repeated scanning for every patient separately in patient's own anatomy in MNI space. Interesting ROIs shown. Every patient has different areas influenced constantly. Areas of CV_MREG_ that corresponded clinical symptoms from patient records are marked accordingly to finding type

All of the DRE patients presented a reproducible CV_MREG_ abnormality (Figure 2) at the individual level. The repeatedly detected CV_MREG_ abnormality was linked to the anatomical area triggering individual seizure manifestation in eight DRE patients (Figure 2). With four patients, there were also other interesting areas (Figure S1). Importantly, no voxels remained after CV_thr_ thresholding in the HC group (Figure 6b).

**Figure 3:**
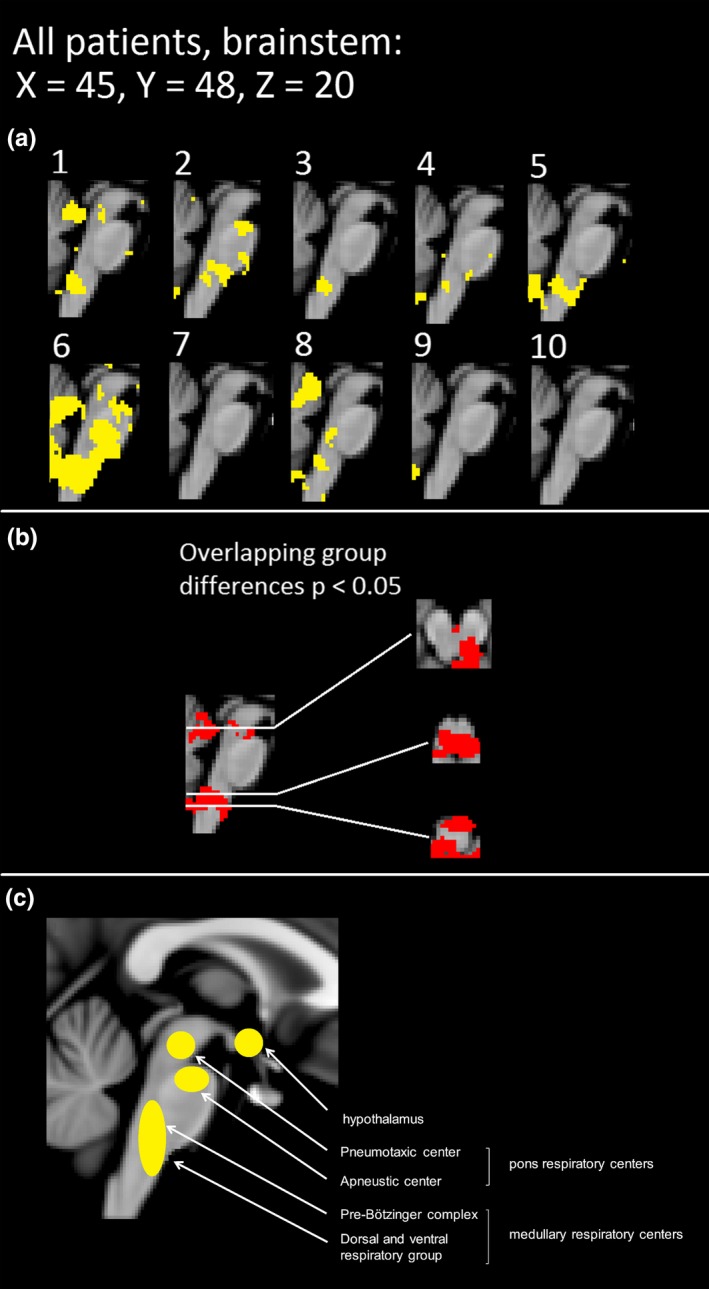
Brainstem alterations on an individual level. (a) Individual threshold CV_MREG_ maps of brainstem alterations in repeated scanning in yellow. (b) The overlapping statistical significant differences between the groups with *p*‐value <0.05 in two scans. Group differences are also displayed in axial view. (c) The anatomical map of brainstem respiration centers. The labeling of anatomical areas was performed in style of the “Fundamentals of Human Physiology,” 4th edition, author: Lauralee Sherwood, Chapter 2, page 374, Fig 12–25

**Figure 4:**
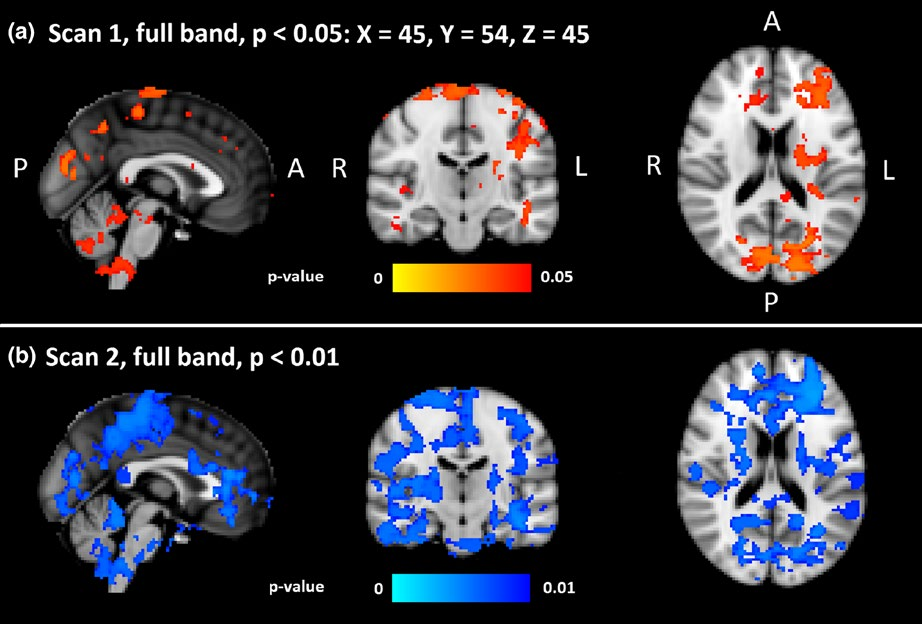
Group level differences. Statistically significant voxels and their *p*‐values in full band fMRI signal between the HC and DRE patients. (a) On the top panel are shown significant differences in the first scan (*p* < 0.05). (b) On the lower panel are shown significant differences of second scan (*p* < 0.01). DRE: drug‐resistant epilepsy

**Figure 5:**
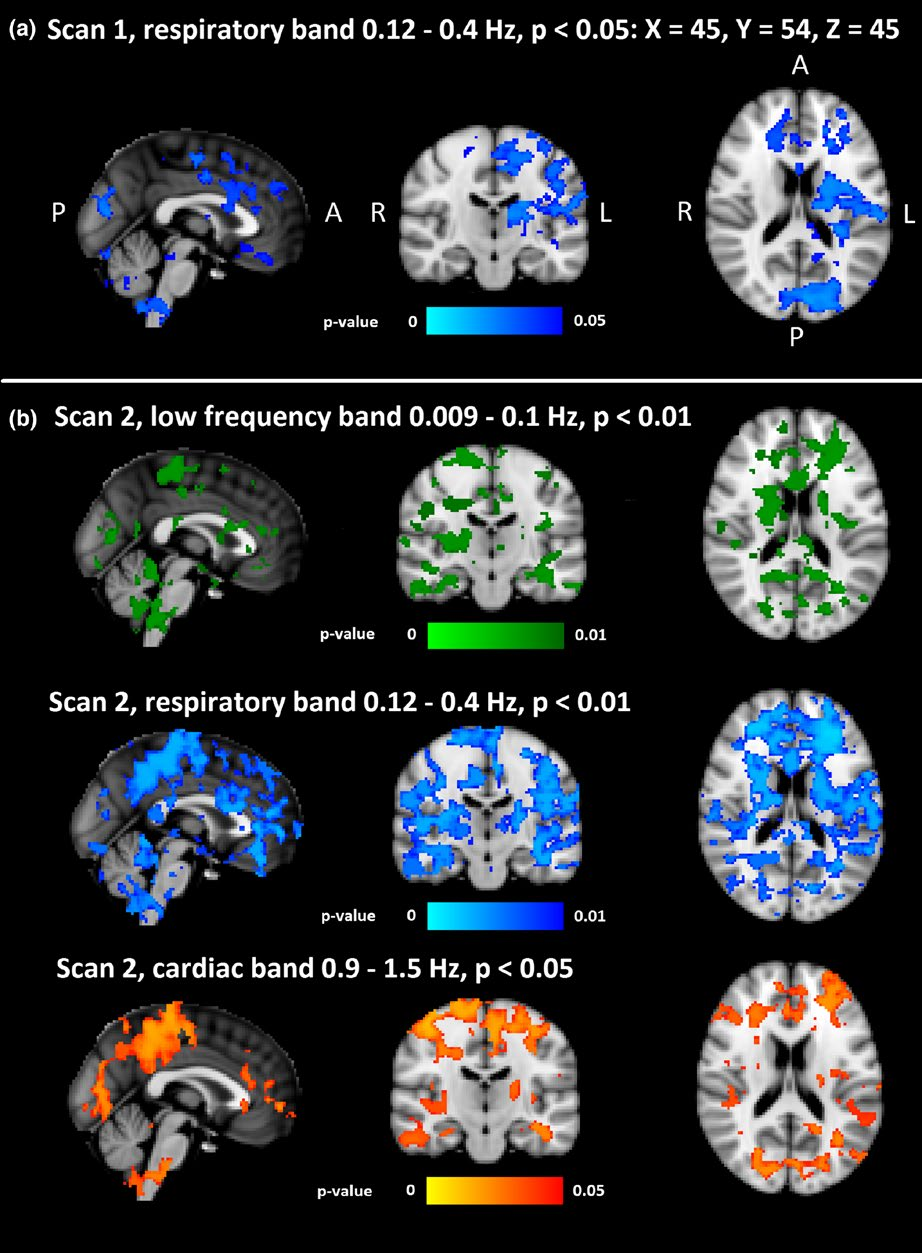
Group level differences in different sub bands. Statistically significant voxels and their *p*‐values in filtered fMRI time series between the HC and DRE patients. (a) In the first scan, there were significant differences (*p* < 0.05) only in respiratory (0.12–0.4 Hz) band. (b) In the second scan, both the low frequency and the respiratory band were significantly different (*p* < 0.01), and the cardiac band was significantly different (*p* < 0.05). DRE: drug‐resistant epilepsy

Subjects with DRE tend to have abnormal autonomous nervous system activity. In terms of autonomous nervous system activity, seven of the 10 DRE patients had altered CV_MREG_ in the brainstem at or near autonomous nervous system nuclei (Figure 3). The individual differences were mostly located in the ventral and dorsal respiratory areas near solitary and ambiguus nuclei, which are part of the reticular activating system responsible for autonomic nervous system control. These areas also showed significant differences in all group analyses (Figures 3,4 and 5).

### 3.2 Spatial CV map differences between groups

The CV_MREG_ of DRE patients was significantly increased compared to HC (*p* < 0.05, Figure 4a). In the full‐band fMRI data, differences were predominantly located in the left hemisphere. Symmetrical changes were detected in primary sensorimotor cortices extending to parietal lobule and to visual cortices V1–V3. Major frontal lobe changes were predominantly located in left cerebral cortex. Additional significant changes were located in left callosal body, the cingulum, in the upper and lower brainstem in ventral and dorsal respiratory centers, and in the cerebelli, where the most dominant areas were left crus I–II, left VII–VIII, vermis, right crus II, right VII–VIII and small lobes in left and right I–IV.

The second measurement showed an increase in the CV_MREG_ difference between patients and controls. At a lowered threshold of *p* < 0.01, extensive differences between DRE patients and HC were observed (Figure 4b). Compared to first measurement, group differences were both more widespread and symmetrical.

### 3.3 Differences between brain pulsation frequencies

Group level analysis of different sub‐bands revealed significant differences in first measurement only in the respiration band (*p* < 0.05, Figure 5a). The second measurement showed group differences in cardiovascular at *p* < 0.05 and in both respiratory and VLF bands at *p* < 0.01 (Figure 5b). Compared to the first measurement, group differences in CV_MREG_ maps of the respiratory band were more widespread in the second measurement.

Also, in the CV_MREG_ maps of second measurement, the respiration band markedly dominated group differences. The VLF changes were localized along the midline and lack the peripheral extent of those changes seen in respiratory band. Interestingly, the differences in the cardiac band included these peripheral cortical structures.

Comparing different sub‐bands of patients’ fMRI CV_MREG_ maps also to secondary verification group (with better physiological data quality with similar results in cardiac PPG and respiratory ETCO_2_ rates during the scanning in both groups, Figure S2) revealed that full‐band CV_MREG_ was even more elevated in DRE patients versus HC (Figure S3).

### 3.4 Template‐based CV‐analysis

**Figure 6:**
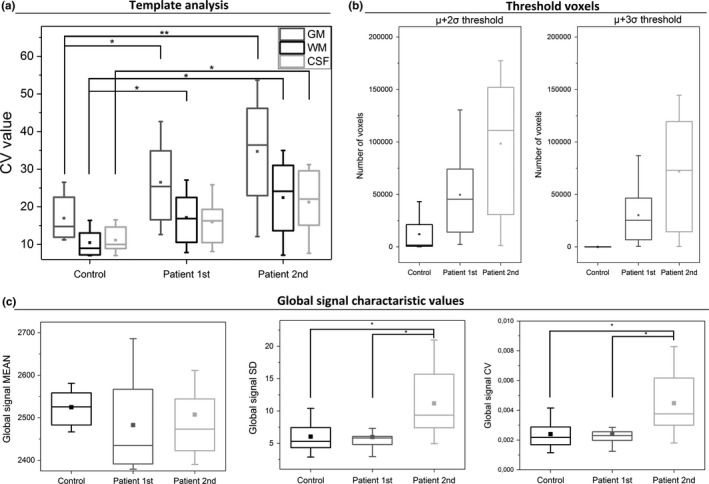
Additional analyze methods between the groups. (a) Template‐based analysis, in which each template voxels CV_MREG_ values are shown. There are statistically significant differences between HC and DRE patients in GM and WM in first scan (*p* < 0.05). In the second scan, the significant difference in GM is more pronounced (*p* < 0.01) than WM and CSF (*p* < 0.05). (b) Interesting voxels left in HC and patient group with *μ*+2σ and *μ*+3σ thresholds. (c) Global signal *μ*, σ, and CV_MREG_ values between groups. There was significant difference (*p* < 0.05) in σ and CV_MREG_ values between HC and second patient scan, but also between first scan and second scan. DER: drug‐resistant epilepsy

Data from both groups indicated that GM has significantly higher CV_MREG_‐values than WM and CSF (Figure 6a). Compared to HC, DRE patients had significantly elevated CV‐values for GM, WM, and CSF in both measurements, while each GM had highest values. Importantly, CV_MREG_ in GM was elevated in the second measurement for every patient.

### 3.5 Thresholded data

Thresholding of the data was performed based on the HC data statistics of the whole brain data. While the HC dataset showed voxels exceeding the >2 *σ* threshold, but not >3*σ* (Figure 6b), maps of DRE patients showed voxels at >3*σ* threshold. The second measurement showed more voxels than the first (Figure 6b). Based on this, a voxel‐wise threshold was applied to the CV_MREG_‐maps.

### 3.6 Global fMRI signal motion and physiological parameters

#### 3.6.1 Global signal

There were no significant differences in mean global signal intensity between groups (Table 2). However, SD_MREG_ and CV_MREG_ of global signal showed a significant difference between the HC group and second DRE patient measurement (Table 2, Figure 6c).

**Table 2:** Global signal and motion parameters between groups

Signal	HC	DRE Scan 1	DRE Scan 2	*p*‐Value: Scan 1	*p*‐Value: Scan 2
Global fMRI: Mean (a.u.)	2,524.80 ± 108.20	2,482.78 ± 106.61	2,507.41 ± 105.43	0.84	0.62
Global fMRI: *SD* (a.u.)	6.04 ± 2.59	5.99 ± 2.20	11.18 ± 5.44	0.45	0.014
Global fMRI: CV	0.0024 ± 0.0010	0.0024 ± 0.0009	0.0045 ± 0.0022	0.55	0.21
Motion: Absolute, mean (mm)	0.94 ± 0.50	1.30 ± 0.25	0.63 ± 0.22	0.14	0.21
Motion: Relative, mean (mm)	0.054 ± 0.019	0.055 ± 0.011	0.048 ± 0.010	0.91	0.34
Motion: Absolute, CV	0.68 ± 0.090	0.73 ± 0.085	0.71 ± 0.061	0.14	0.68
Motion: Relative, CV	0.77 ± 0.15	0.90 ± 0.19	0.66 ± 0.10	0.14	0.10

*Note*

DRE: drug‐resistant epilepsy.

#### 3.6.2 Motion

**Figure 7:**
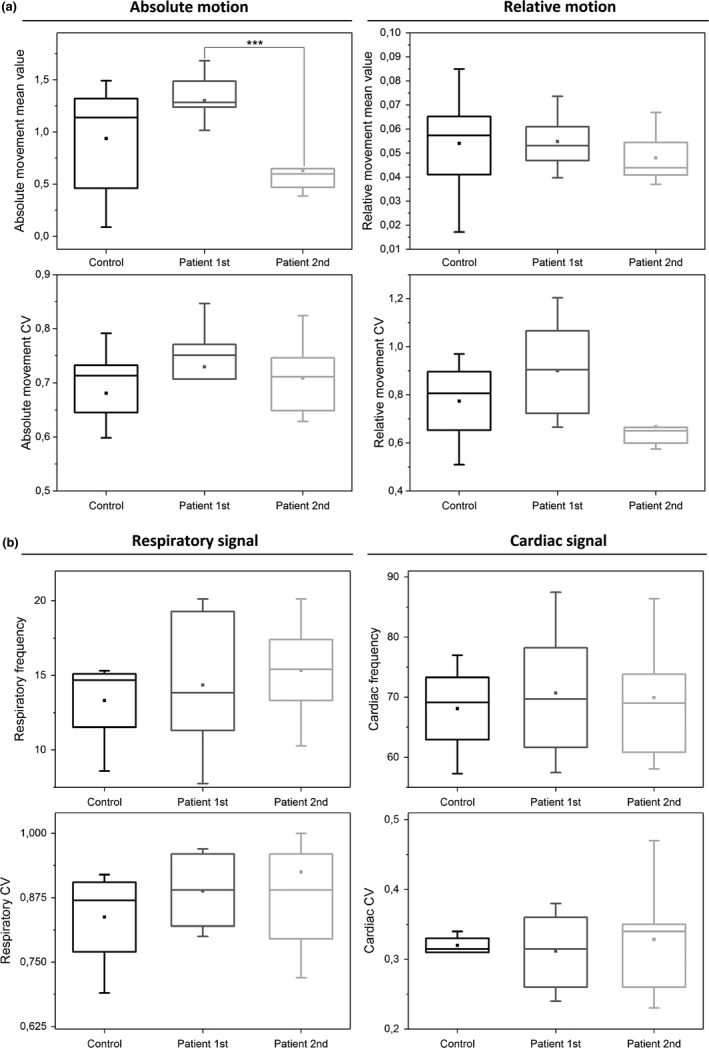
Motion and physiological parameters. (a) Absolute and relative motion and their CV_MREG_ values between groups. Only difference is that there is much less absolute motion in second scan (*p* = 0.00044). (b) Differences in both respiratory and cardiorespiratory signals between groups were absent

There were no differences in motion parameters between the HC group and the DRE patients (Table 2, Figure 7a) Furthermore, neither CV_Motion_ of absolute nor relative movement of time domain motion signal were significantly different between groups (Table 2, Figure 7a). The only significant difference was a *reduction* of the absolute movement in the DRE patients second measurement compared to the first (*p* = 0.00044).

#### 3.6.3 Cardiorespiratory data

For physiological analysis, six controls and two DRE patients lacked physiological data and were excluded with two matching individuals. In the remaining data, there were no statistically significant differences between groups neither in heart nor respiration (Table 3, Figure 7b). Due to lack of some physiological data in matched HC, we also used a secondary verification HC_2nd_ group having a better quality physiological data measured identically synchronous with fMRI. In the secondary control group, one respiration belt and one pulse oximetry dataset were still of bad quality. The HC_2nd_ group did not have significant differences compared to DRE group either (Table 3, Figure 7b). Neither the CV of the heart nor respiration rates were significantly different between HC/HC_2nd_ versus DRE patients (Table 3, Figure 7b).

**Table 3:** Cardiorespiratory signal characteristics between groups

Signal	HC	DRE combined scans	*p*‐Value
Cardio: frequency	68.11 ± 8.14	70.29 ± 9.90	0.70
Cardio: CV	0.32 ± 0.014	0.32 ± 0.066	0.93
Respiratory: frequency	13.31 ± 3.16	14.92 ± 3.75	0.74
Respiratory: CV	0.84 ± 0.10	0.91 ± 0.16	0.50
Cardio, HC_2nd_: frequency	71.46 ± 10.26		0.80
Cardio, HC_2nd_: CV	0.30 ± 0.030		0.47
Respiratory, HC_2nd_: Frequency	16.56 ± 3.08		0.32
Respiratory, HC_2nd_: CV	0.81 ± 0.20		0.45

*Note*

DRE: drug‐resistant epilepsy.

## 4 DISCUSSION

Our results indicated an altered variation of physiological brain signals in DRE patients. The group differences consisted of highly significant alterations in CV_MREG_, more precisely in the SD_MREG_ of the time domain signal. The altered signal variability dominated in VLF and respiratory bands. All patients showed individual but robust changes in the physiological noise CV_MREG_ >3σ above normal HC mean in two measurements. The increased variation was not explained by any of the multimodal cardiorespiratory control measures nor by rigid body head motion differences between HC versus DRE patients. Interestingly, in the 2nd fMRI measurement, DRE patients exhibited reduced absolute motion but increased physiological signal variation.

### 4.1 Individual thresholded diagnostic mapping of abnormal physiological signal in epilepsy

To the best of our knowledge, this is the first time that an fMRI signal variation has been used to show significant individual brain signal changes in DRE patients in the absence of epileptic activity. DRE patients showed repeating increases in physiological variation of the fMRI signal measured as CV_MREG_ and band passed *SD*. On the group level, changes in CV_MREG_ showed widespread increases in fMRI signal variation against *two* control groups (one being age and sex matched and the other with verifiably more similar physiological pulsation data).

On the individual level, these changes were confined to large anatomical areas in majority of cases and in three cases to a sub‐centimeter frontal foci. This suggests that CV_MREG_ may enable subject‐specific mapping of case‐specific changes. Previously, due to limited statistical power of slowly sampled fMRI data, it was not feasible to threshold DRE patients’ CV_MREG_ maps at individual level with the healthy control population threshold of 3 *SD*. Since we do not find CV_MREG_ changes >3 *SD* in the control groups, we feel that CV_MREG_ may be a marker at individual level.

The reason for the increased sensitivity stems from the ultra‐fast fMRI image sampling as it removes several major confounds that have previously prevented comprehensive analysis of human brain physiology in fMRI. First of all, the critical signal sampling of physiological brain pulsations prevents aliasing of these pulsations on top of each other, which then enable clear distinction between the physiological phenomena after band pass. Secondly, the prompt 80‐ms acquisition of the k‐space in MREG captures the spreading pulse effects accurately, while commonly applied interleaved slice sampling mixes the propagation due to 1,3,5…2,4,6 timing of slices. Thirdly, the statistical power of fast‐fMRI is elevated due to 20‐fold increase in samples. Previously, these advances enabled separation of *single* epileptic spikes in an EEG‐MREG study (Jacobs et al., 2014). The CV analysis of signal variation benefits from more accurate and statistically powerful data and thus enables the formation of individual CV_MREG_ maps, when thresholded against values 3*σ* above normal level.

Combined EEG‐fMRI usually markedly improves the fMRI mapping of the epileptic activity (Moeller et al., 2009), with the requirement of the activity to be present during the multimodal scanning. However, our patients did not exhibit epileptic activity. Fast fMRI CV_MREG_‐maps showed novel information at individual level regarding the brain pathophysiology in DRE that matched overall clinical assessment in majority of cases.

### 4.2 Group results

Both mesial temporal lobe and idiopathic generalized epilepsy have shown increased amplitude of low‐frequency BOLD signal fluctuations in thalami, temporal cortices, and default mode network (DMN) (Wang et al., 2014; Zhang et al., 2010). It is also shown that there might be relation between VLF differences on white matter and BOLD signal detection (Gawryluk, Mazerolle, & D'Arcy, 2014). The epilepsy patients’ functional connectivity of resting state BOLD signal also reduced within DMN, and between epileptic areas and DMN (Constable et al., 2013; J. Gotman et al., 2005; Lee, Smyser, et al., 2013; Robinson et al., 2017). Zhang et al. (2015), have found that a combined measure of BOLD signal amplitude and functional connectivity density is abnormal in epilepsy. However, recent studies showed both increased and decreased changes in BOLD amplitude and connectivity measures (Centeno & Carmichael, 2014; Robinson et al., 2017). Our previous evidence in resting state fMRI also showed both increases and decreases in regional and long‐range connectivity measures in epilepsy (Mankinen et al., 2011, 2012).

The group level results indicated *only* increased variation in the fMRI signal in DRE patients. The group level changes were widespread, especially in the respiratory and VLF bands. As the underlying signal characteristics were altered in DRE, they were bound to have an influence on interleaved BOLD data with 2‐ to 3‐s sampling rate. The increased physiological noise can further alias over low frequency in interleaved echo‐planar imaging BOLD signal of 2–3 s in spurious ways, and some of the previous mixed results can be explained by differential aliasing of physiological pulsations in the brain (Kiviniemi, Kantola, Jauhiainen, & Tervonen, 2004).

### 4.3 Pathophysiological mechanism behind pulsation abnormality

The recently discovered glymphatic brain clearance system uses physiological pulsations to clear the brain tissue by convecting water through the brain tissue via aquaporin AQP4 water channels especially during sleep (Iliff & Nedergaard, 2013; Jessen, Munk, Lundgaard, & Nedergaard, 2015; Nedergaard, 2013). In human brain, glymphatic system may be driven partially by respiratory and vasomotor control waves in addition to cardiac pulses (Kiviniemi et al., 2016).

The most common cause in DRE is MTS, which causes 40%–50% of all DRE cases (Jutila et al., 2002; Lapalme‐remis & Cascino, 2016). Tissue samples of MTS have been shown to present a striking lack of peri‐vascular AQP4 water channels that mediates the glymphatic water clearance (Eid et al., 2005). The absence of AQP4 channels in MTS areas could lead to altered extracellular electrolyte concentrations that sensitizes neuronal tissue to seizures (Eid et al., 2005; Lundgaard et al., 2017; Marchi et al., 2007).

The results of our study suggest that the physiological pulsations, that drive glymphatic clearance, may be abnormal in drug‐resistant epileptic brain (Kiviniemi et al., 2016; Nedergaard, 2013). As none of our DRE patients had MTS lesions, we suggest that seemingly normal DRE patients’ brain tissue may suffer from failed physiological drive of the glymphatic clearance, rather than direct AQP4 channel loss as in MTS. The pulsation abnormality becomes evident as abnormal signal variation in the specific physiological frequency bands, especially respiratory and very low frequencies. These frequencies further overlap with autonomic disturbances known to be present in epilepsy (Ansakorpi et al., 2002, 2011; Liu et al., 2017; Suorsa et al., 2011; van der Kruijs et al., 2016).

Furthermore, the pulsation abnormalities could be linked to the detected abnormalities in neurovascular coupling in epilepsy, where an abnormal and prolonged dilation in the blood vessels are detected prior to epileptiform activity (Jacobs et al., 2009; Mäkiranta et al., 2005; Moeller et al., 2009; Osharina, Aarabi, Manoochehri, Mahmoudzadeh, & Wallois, 2017). Prolonged vasodilatations may minimize glymphatic pulsation of the perivascular CSF reservoir and lead to altered tissue homeostasis and further to epileptic activity (Jessen et al., 2015).

The VLF (0.01–0.1 Hz) and LF (i.e., respiratory 0.3 Hz) pulsations bands have previously revealed autonomous nervous system changes in heart rate variability (Ansakorpi et al., 2002, 2011; Liu et al., 2017; Lotufo, Valiengo, Benseñor, & Brunoni, 2012; Suorsa et al., 2011; van der Kruijs et al., 2016). The abnormal, noncoupled vasodilation may be linked to the detected VLF vasomotor pulsation increase and to the detected noise changes in the brainstem autonomous nervous system centers (Figure 3). The previous studies have also shown that connectivity of the brainstem and cortical/subcortical structures is altered in temporal lobe epilepsy (Englot et al., 2017). The LF power changes were mapped to brainstem areas in seven of 10 patients in our study indicating high probability of dysregulatory changes in these areas. Our results indicated that fast fMRI offers new diagnostic information on the neurovascular control also at brainstem level.

### 4.4 Sources of the signal features

In this study, we showed for the first time abnormally increased fMRI signal variation in DRE linked to the *intrinsic* physiological pulsatility of the brain. While the external markers of cardiovascular physiology and rigid body motion showed no differences compared to two control groups, the brain pulsations in these frequencies still are highly abnormal in DRE. Coefficient of variation reflects stability of the measured signal and increased motion of the subject increases CV (Hajnal et al., 1994; Hao, Khoo, von Ellenrieder, & Gotman, 2017). Also, in this study, the first measurement of the DRE patients had more absolute motion compared to HC. And indeed, in the first measurement, the CV_MREG_ was found to be increased in the fMRI signal of the DRE patients. In the second measurement, however, where patients’ absolute movement was reduced, CV_MREG_ was even more increased compared to HC. Therefore, motion does not explain our results. We interpret that in the case of CV_MREG_ mapping, which is sensitive to any noise source, increased motion may mask the pathological brain tissue CV_MREG_ changes, since in the absence of motion, the differences become highly significant. We assume that this reduction in absolute movement could be linked to patient habituation to the scanning and verified that it did not affect the CV_MREG_ results by comparing the control group data.

A recent finding regarding the global fMRI signal *SD* indicates that indeed the arousal of the subjects may have a widespread effect on the fMRI results (Chang et al., 2016; Liu et al., 2018). Subjects with epilepsy have altered sleep homeostasis and present widespread and complex neural and hemodynamic signal changes that are different in deep brain structures compared to cortex (Boly et al., 2017; Peter‐Derex, Magnin, & Bastuji, 2015; Salek‐Haddadi et al., 2003). Thus, DRE changes induced by altered arousal mechanisms might explain the differences in 1st vs. 2nd scans. As many subjects may fall asleep after only a few minutes, the second scan may have reduced vigilance that increase sensitivity to abnormal CV values (Tagliazucchi & Laufs, 2014). Furthermore, drowsiness could explain the detected reduction in the motion of the DRE, which was the only difference in motion in the groups. An interesting future project would be investigation of sleep and vigilance fluctuations in combined EEG‐fMRI data.

None of the physiological measures showed differences between the groups, which minimizes the possibility of a direct physiological noise source difference. The datasets were rigorously corrected with conventional FSL preprocessing and with advanced ICA‐based FIX that further de‐noises the data for motion and other noise sources (Griffanti et al., 2014; Salimi‐Khorshidi et al., 2014). Without FIX, there were no differences between the HC and first measurement of epilepsy. Again, in the second measurement with less motion artifact, even the raw data had increased CV_MREG_ in DRE patients (Figure S2).

### 4.5 Limitations and future prospects

Medication may cause some of the detected signal changes, but preliminary data from drug‐naïve epilepsy patients showed converging results (Figure S4), so the found effect is not only seen in DRE, but also with newly diagnosed drug‐naïve epilepsy patients. The analysis would naturally benefit from a higher sample size and second measurement verification data also for controls, which are under way. We plan to generate a new CV_MREG_ threshold image with more controls subjects, and for this aim, we are creating an international imaging cluster for speeding up data acquisition of healthy control subjects.

As CV has been successfully used in various blood flow‐related signal measurement techniques in different scales from mouse microscopy to macroscopic human data (Jahanian et al., 2014; Kalchenko, Kuznetsov, Meglinski, & Harmelin, 2012; Makedonov et al., 2013, 2016), it would be interesting if one could use it in multimodal imaging as a way to produce scale invariant measures that could combine animal model data to clinical human brain data.

## 5 CONCLUSIONS

Our statistical analysis revealed highly significant evidence for discarding of the null hypothesis of equal physiological noise. We further evaluated the contribution of the three basic physiological pulsation bands (cardiac, respiratory, and vasomotor <0.1 Hz frequencies) as a source of CV noise change. The changes in brain CV_MREG_ in the absence of diagnostic epileptic discharges in DRE patients were not explained by motion or measured cardiorespiratory rates. After thresholding the CV_MREG_ values based on control data, we were able to identify repeating increases in individual DRE patient's CV_MREG_ maps 3 standard deviations above mean.

CV mapping of fast fMRI signal revealed a marked increase in intrinsic physiological brain signal variation in DRE patients. Neither external measures of cardiorespiratory activity, electroencephalography nor bulk head motion, explained the increased CV_MREG_ and therefore the changes are most likely due to intrinsic brain pulsations. Abnormally high CV_MREG_ noise values 3 *SD* above HC mean were only detected in patients, which render the use of CV_MREG_ to individually map altered brain pulsation areas possible in epilepsy. The most dominant altered frequencies were in the respiration/parasympathetic 0.12–0.4 Hz range and very low frequency range (<0.01 Hz) with changes in brainstem areas controlling autonomous system in majority of cases. The results indicate a possibility for a novel diagnostic tool for a functional MRI signal in DRE.

Previous findings of the most common cause of DRE, that is, MTS have shown glymphatic aquaporin AQP4‐channel absence from perivascular astrocytes. Our results showed marked alterations in physiological pulsations that drive the glymphatic clearance without any signs of MTS in DRE. Our findings indicate that the glymphatic pulsation mechanisms may suffer from failure of glymphatic function in DRE, leading to altered tissue homeostasis that promotes seizures development. Thus, a pathological alteration of the glymphatic clearance mechanisms explains the failure of all modern medications in DRE that are usually targeted to electrolyte channel blockage rather than solute clearance.
