# Supplementary figures and images for: Altered physiological brain variation in drug‐resistant epilepsy
Source: Brain Behav. 2018 Aug 15;8(9):e01090. doi: 10.1002/brb3.1090 (PMC6160661; doi:10.1002/brb3.1090)

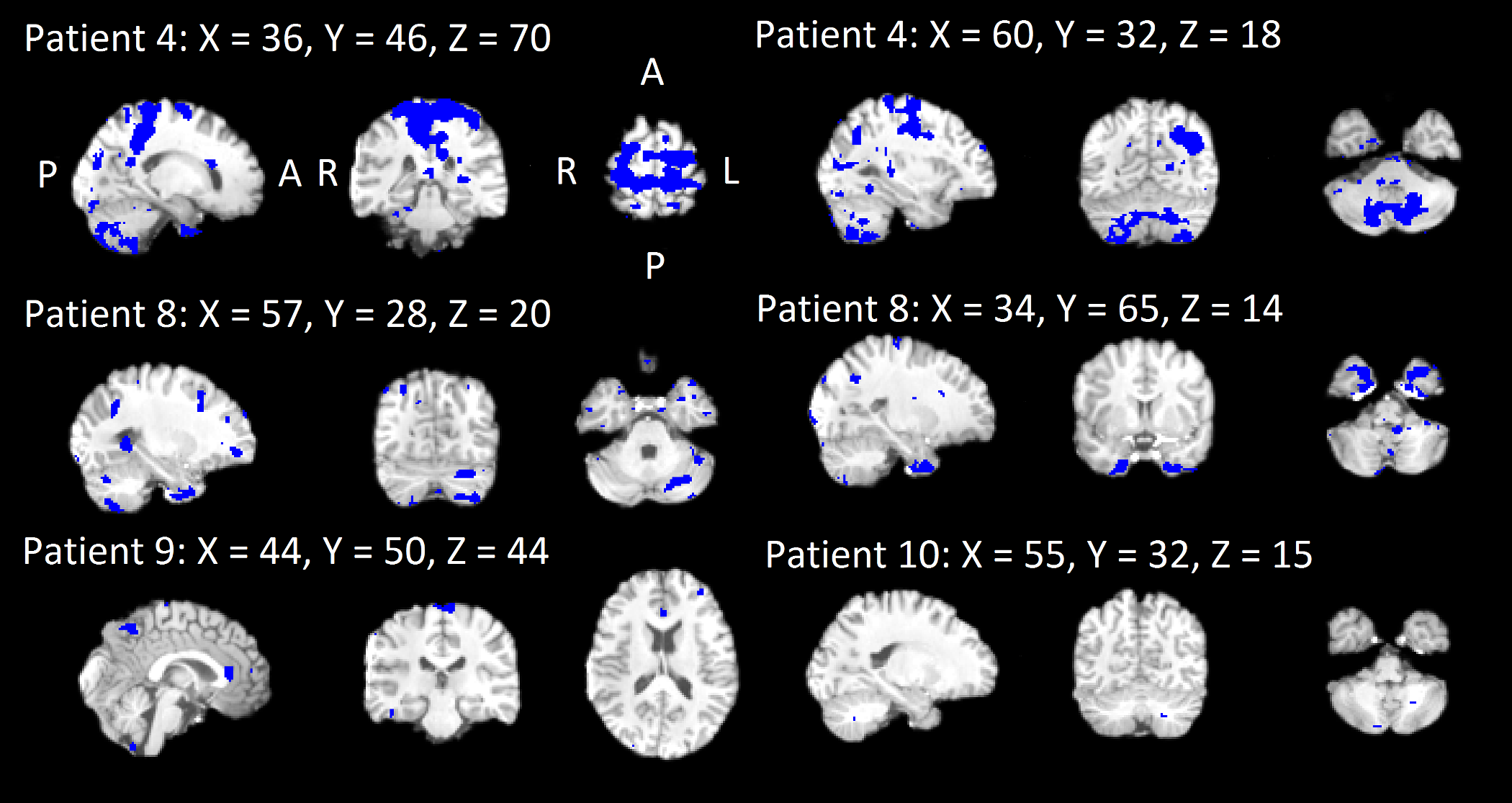

Supplement: Supplementary file 1 [file BRB3-8-e01090-s001.tif]

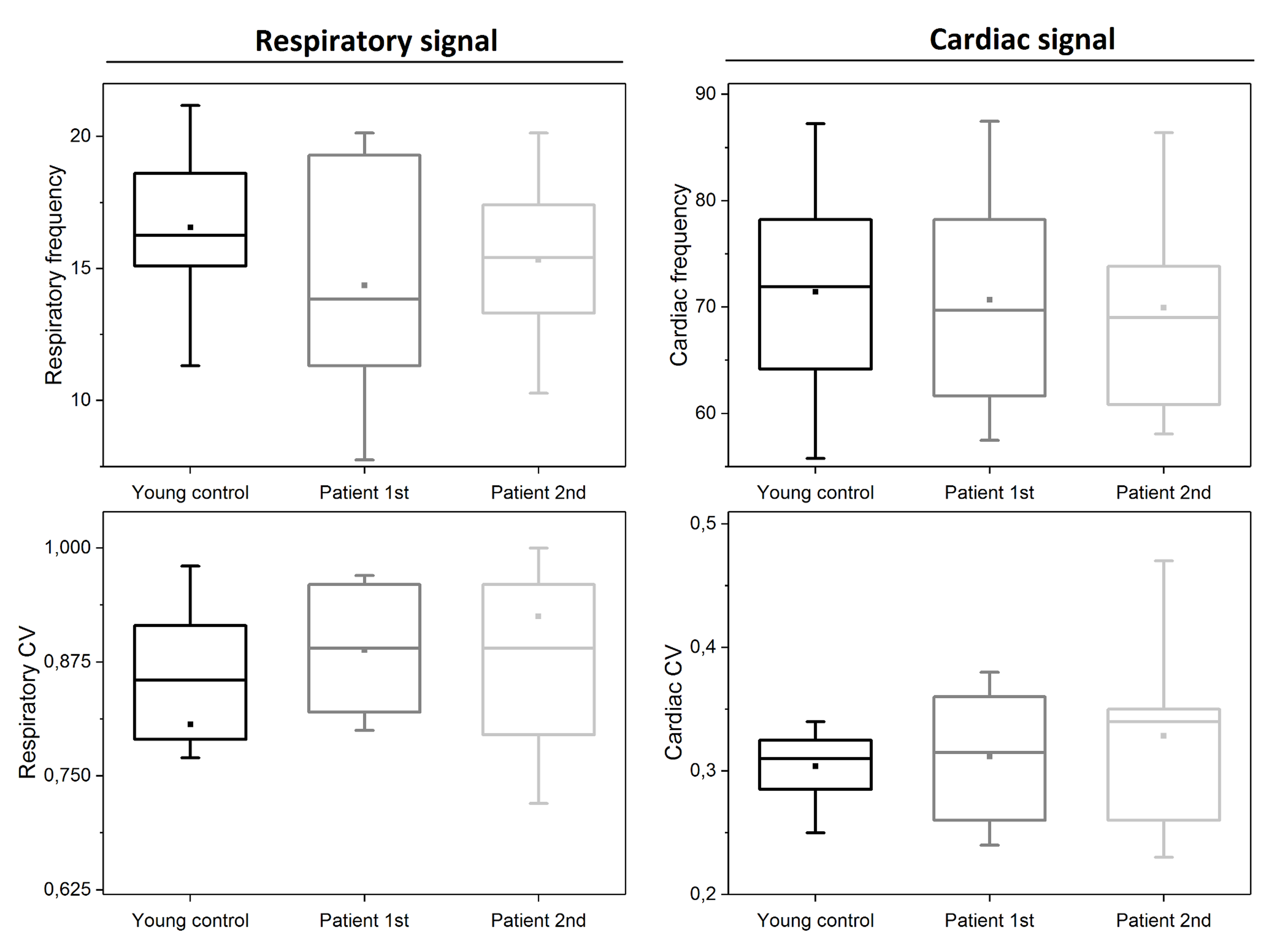

Supplement: Supplementary file 2 [file BRB3-8-e01090-s002.tif]

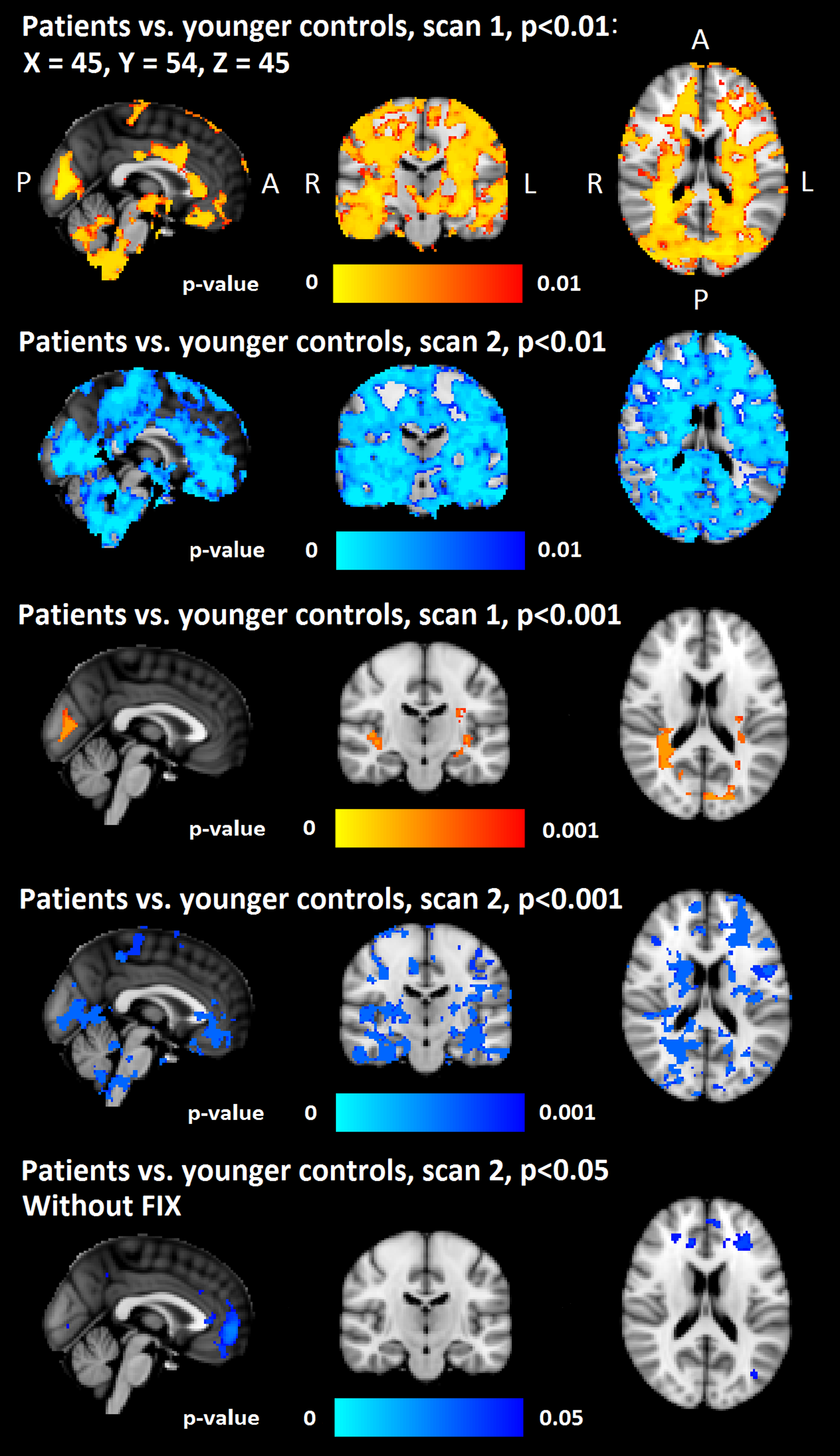

Supplement: Supplementary file 3 [file BRB3-8-e01090-s003.tif]

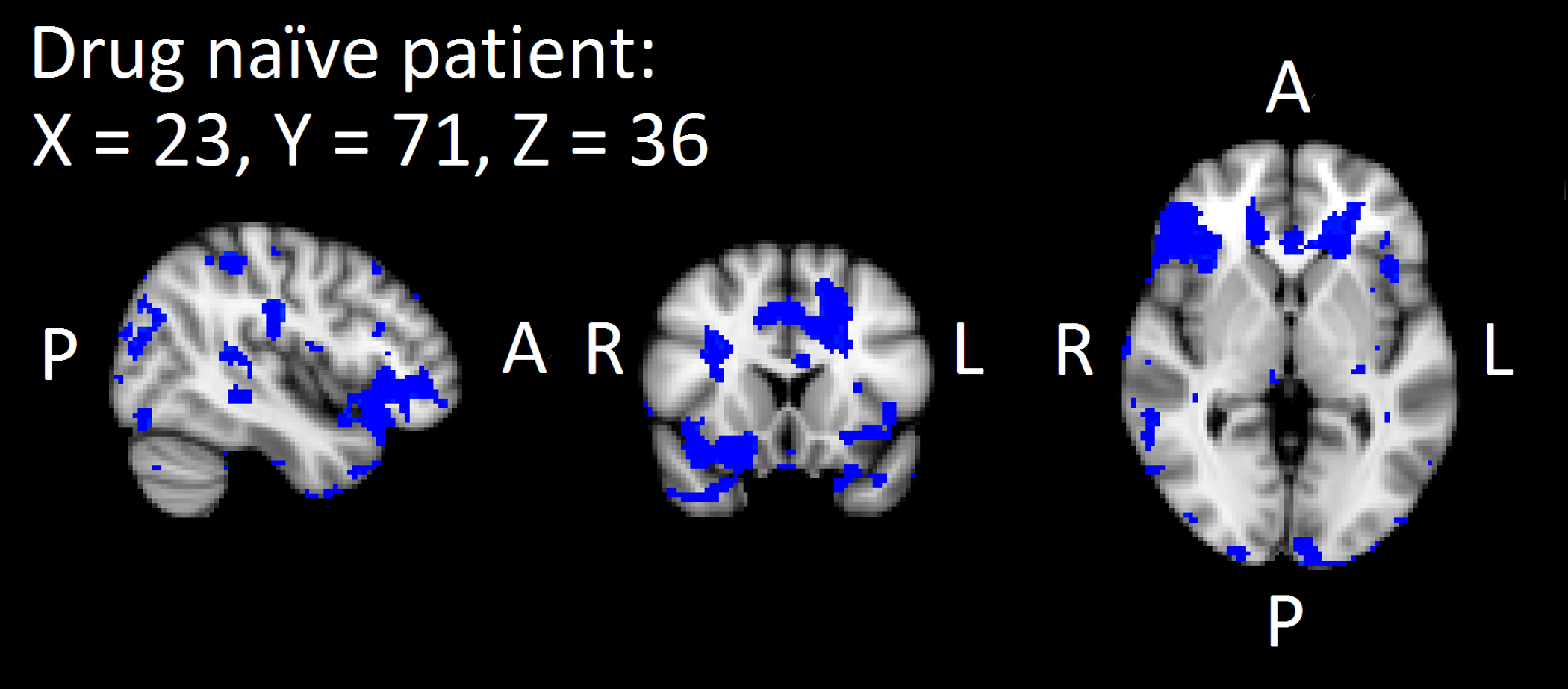

Supplement: Supplementary file 4 [file BRB3-8-e01090-s004.tif]
